# Real-world outcomes of patients with hereditary angioedema with normal C1-inhibitor function and patients with idiopathic angioedema of unknown etiology in Canada

**DOI:** 10.1186/s13223-024-00910-x

**Published:** 2024-09-27

**Authors:** Adil Adatia, Jean-Nicolas Boursiquot, Dawn Goodyear, Chrystyna Kalicinsky, Amin Kanani, Susan Waserman, Michelle M. L. Nguyen, Abhinav Wadhwa, Jessica Weiss, Ahmed El-Zoeiby, Stephen Betschel

**Affiliations:** 1 Alberta Health Services, University of Alberta, Edmonton, AB Canada; 2 CHU de Québec, Université Laval, Québec, QC Canada; 3 Alberta Health Services, University of Calgary, Calgary, AB Canada; 4 Health Sciences Centre, University of Manitoba, Winnipeg, MB Canada; 5 St. Paul’s Hospital, University of British Columbia, Vancouver, BC Canada; 6 Hamilton Health Sciences, McMaster University, Hamilton, ON Canada; 7 Pentavere Research Group Inc., Toronto, ON Canada; 8 Takeda Canada Inc., Toronto, ON Canada; 9 Unity Health Toronto, St Michael’s Hospital, University of Toronto, Toronto, ON Canada

**Keywords:** Hereditary angioedema, Real-world evidence, Treatment, Outcomes, Normal C1 inhibitor

## Abstract

**Background:**

Hereditary angioedema with normal C1-inhibitor function (HAE nC1-INH) and idiopathic angioedema of unknown etiology (AE-UNK) are rare conditions that cause recurrent subcutaneous and submucosal swelling. The characteristics and clinical outcomes of patients with these conditions in Canada have not been studied.

**Methods:**

The aim of this study was to extract real-world evidence from the electronic health records of patients with HAE nC1-INH or AE-UNK who were managed in selected practices of Canadian HAE-treating specialist physicians between 01-Jan-2012 and 01-Jan-2022, to examine case numbers, treatment, clinical outcomes, and healthcare utilization.

**Results:**

Of 60 patients (37 with HAE nC1-INH, 23 with AE-UNK), median (range) age at symptom onset was 21.5 (5.0–57.0) and 23.0 (10.0–54.0) years, respectively. Time to diagnosis from onset of symptoms was 7.0 (0.0–43.0) and 2.0 (− 10.0 to 50.0) years. Significant differences were observed in terms of the predominant triggers for angioedema attacks between patients with HAE nC1-INH and AE-UNK: stress (65% vs. 26%, p = 0.007) and estrogen therapy (35% vs. 9%, p = 0.031). Before diagnosis, most patients received antihistamines (50% of HAE nC1-INH and 61% of AE-UNK patients). Post-diagnosis, 73% and 74% of HAE nC1-INH and AE-UNK patients received long-term prophylaxis (LTP), with the most common LTP treatments being subcutaneous pdC1-INH (43% of HAE nC1-INH patients and 39% of AE-UNK patients) and tranexamic acid (41% of HAE nC1-INH patients and 35% of AE-UNK patients). Of patients with HAE nC1-INH, and patients with AE-UNK, 22% and 13%, respectively, were taking more than one LTP treatment concurrently. Before HAE treatment initiation, significantly fewer patients with AE-UNK compared to patients with HAE nC1-INH had angioedema attacks affecting their extremities (13% vs. 38%, p = 0.045) and GI system (22% vs. 57%, p = 0.015). In the three months following treatment initiation, patients with AE-UNK experienced significantly fewer angioedema attacks compared to patients with HAE nC1-INH (median 2.0 attacks [0.0–48.0] vs. 6.0 attacks [0.0–60.0], p = 0.044). Additionally, fewer patients with AE-UNK compared to HAE nC1-INH experienced attacks affecting their GI system (26% vs. 57%, p = 0.032). Attack duration and frequency significantly decreased for patients with HAE nC1-INH from a median of 1.00 day (range: 0.00–7.00) to 0.29 day (range: 0.02–4.00; p = 0.001) and from 10.50 attacks (range: 0.00–90.00) to 6.00 attacks (range: 0.00–60.00; p = 0.004) in the three months following HAE treatment initiation.

**Conclusions:**

Using Canadian real-world evidence, these data demonstrate differing clinical trajectories between patients with HAE nC1-INH and AE-UNK, including diagnostic delays, varied attack characteristics, treatment responses and healthcare utilization. Despite treatment response, many patients still experienced frequent angioedema attacks. These results suggest an unmet need for treatment guidelines and therapies specifically for patients with HAE nC1-INH and AE-UNK and better understanding of the pathophysiology accounting for the reported differences between the two.

**Supplementary Information:**

The online version contains supplementary material available at 10.1186/s13223-024-00910-x.

## Background

Hereditary angioedema (HAE) is a chronic and rare disease characterized by recurrent and unpredictable episodes of swelling, predominantly in the subcutaneous and/or submucosal tissues of the extremities, larynx, face, abdomen, and genitals [1, 2].

HAE can be categorized into two main types: HAE due to C1 inhibitor deficiency (HAE-C1-INH) and HAE with normal C1 inhibitor (HAE nC1-INH). HAE-C1-INH is due to an absolute or functional deficit of C1 inhibitor (Type I and Type II, respectively). In contrast, HAE nC1-INH presents with normal serum concentrations and function of C1 inhibitor, but similarly causes recurrent angioedema that is refractory to antihistamines and glucocorticoids [3]. Six monogenic forms of HAE nC1-INH have been described, but in most patients a genetic etiology is not identified [4]. Additionally, there are patients with recurrent non-histaminergic angioedema and normal C1 inhibitor who do not have a family history of similar disease, which can be termed idiopathic angioedema of unknown etiology (AE-UNK) [5, 6].

HAE nC1-INH and AE-UNK appear to be far less prevalent than HAE C1-INH [3, 7, 8], but there are few epidemiologic studies assessing their frequency [8] In Germany, case numbers of HAE nC1-INH were estimated to be 1:100,000 in 2015 and 1:200,000 in Manitoba, Canada in 2019 [9, 10], based on cases identified and the population of Germany and Manitoba respectively, and for AE-UNK they were estimated to be 1:200,000 in Manitoba, Canada in 2022 [5].

Clinical care and research in HAE nC1-INH and AE-UNK are severely hindered by the lack of confirmatory diagnostic tests and limited genetic testing which are collected. Presently the diagnosis of HAE nC1-INH (in the majority without an identifiable genetic cause) and AE-UNK relies on the careful exclusion of alternate causes by a specialist physician typically by therapeutic trials of antihistamines, glucocorticoids, and anti-IgE biologic therapy. This process leads to long delays in diagnosis and difficulties in accessing treatment for patients, and impedes the development of novel therapeutic agents [5, 8].

There are no approved treatments for HAE nC1-INH or AE-UNK. Based on observational studies and case reports, however, on demand (e.g., icatibant and plasma-derived C1 inhibitor [pdC1-INH] and long-term prophylaxis treatments (e.g., lanadelumab, pdC1-INH, and berotralstat) for HAE-C1-INH may be effective in some patients with HAE nC1-INH or AE-UNK [1, 11, 12]. Development of such treatments for HAE nC1-INH and AE-UNK requires improved characterization of these patient populations to understand the burden of disease and clinical outcomes.

In Canada, patients with HAE are primarily treated by allergists or immunologists. During routine clinical care, these clinics collect considerable real-world evidence (RWE) in the form of structured data (e.g., hospital pharmacy orders) and unstructured data (e.g., clinical notes), which is collected and stored in Electronic Health Records (EHR). RWE from EHR can be utilized to better understand disease prevalence, treatment patterns and clinical outcomes over time, and is particularly important for rare diseases for which patient numbers are small and clinical trials are lacking.

The aim of this study was to extract RWE from the EHR of patients with HAE nC1-INH and AE-UNK from selected practices of Canadian HAE-treating specialist physicians, to better understand the case numbers, treatment use, clinical outcomes, and healthcare resource utilization among patients with HAE nC1-INH or AE-UNK, in Canada.

## Methods

### Study design

This was a retrospective cohort study of data from EHR stored at six Canadian practices in five Canadian provinces on HAE patients managed by HAE-treating specialist physicians, between January 1, 2012, and January 1, 2022. All patients who were ≥ 12 years of age with a diagnosis of HAE nC1-INH or AE-UNK were included in the study. Diagnosis of HAE nC1-INH or AE-UNK was based on the following criteria:

1. Recurrent angioedema as documented by a healthcare professional in the specialists charts.
2. Normal C4.
3. Normal C1 level and function.
4. Lack of response to corticosteroid or regular, high-dose and/or prophylactic antihistamine(s) treatment(s).

Additional, but not essential criteria:

1. Condition worsened with estrogen if estrogen is/was received.
2. Family history of non-histaminergic angioedema for patients with HAE nC1-INH.

Patients were excluded if they had other types of angioedema (HAE Type I, HAE Type II, acquired angioedema, etc.), experienced a response to treatments used for histamine-related angioedema or did not meet the diagnostic criteria for inclusion. Follow-up data from EHRs was included up to the extent that they were available within the study period.

### Data extraction

Patient characteristics, treatment use, clinical outcomes and healthcare resource utilization were extracted directly from patients’ EHR and uploaded into an electronic case report form (eCRF) specifically designed for this study. Data was extracted by HAE-treating specialist physicians or their trained staff. HAE-treating specialist physicians were required to review and approve all eCRFs entered by their staff. Data were independently extracted at each site without inter-site discussions during the extraction process. This approach was taken to ensure that the data collected accurately reflected the individual practices of each HAE-treating specialist.

### Outcomes

The primary outcome of interest was:

1. The number of patients in each practice diagnosed with HAE nC1-INH or AE-UNK.

Secondary outcomes of interest included:

1. Treatment use, including: type, dose, duration and setting of treatments received by each patient, treatment combinations in patients receiving multiple LTP therapies.
2. Clinical outcomes, including: attack characteristics before and after treatment initiation and time from treatment (on demand therapy) to symptom improvement/resolution.
3. Healthcare utilization, including: number of primary care visits, number of walk-in visits per year, number of unscheduled physician visits (not including ER visits) per year, number of emergency room visits per year, number of hospitalizations per year, and number of intubations per year.

### Statistical analyses

Descriptive analyses were performed to summarize patients’ characteristics, treatment use, clinical outcomes, and healthcare utilization. Continuous variables were described using mean and standard deviation (SD), and median and the range. Categorical variables were described by frequencies and related percentages. Number of missing observations were reported for all variables.

Exploratory analyses were conducted to identify patterns or potential differences in patient characteristics and/or outcomes between patients with HAE nC1-INH and AE-UNK. For continuous variables, comparisons were conducted using the Wilcoxon rank-sum test. Discrete variables were analyzed using Fisher’s exact test, due to sample size. Comparison of clinical outcomes before and after receiving treatment were performed using the Wilcoxon signed-rank test (for paired, continuous data) and McNemar’s test (for paired, categorical data). Statistical significance was set at p < 0.05.

## Results

### Patients

Between 01 January 2012 and 01 January 2022, 60 patients (37 HAE nC1-INH, AE-UNK) were identified in six clinics across five provinces in Canada. Patients with HAE nC1-INH and AE-UNK had a median (range) age at symptom onset of 21.5 (5.0–57.0) and 23.0 (10.0–54.0) years, respectively (Table 1). The median age at diagnosis for patients with HAE nC1-INH was 35.0 (12.0–73.0) and 32.5 (16.0–80.0) years for patients with AE-UNK (Table 1). Median (range) time to diagnosis from the onset of symptoms was 7.0 years (0.0–43.0) for patients with HAE nC1-INH and 2.0 years (− 10.0 to 50.0) for patients with AE-UNK (Table 1). Prior to diagnosis, the most received angioedema therapy was antihistamines for patients with HAE nC1-INH (59%) and for patients with AE-UNK (61%; Table 1).

**Table 1:** Baseline clinical and demographic characteristics

	HAE nC1-INH (N = 37)	AE-UNK (N = 23)
Sex		
Female	31 (84%)	19 (83%)
Male	6 (16%)	4 (17%)
Age at first symptoms/attack		
N	28	13
Mean (SD)	26.18 (15.89)	28.38 (16.38)
Median (range)	21.50 (5.00, 57.00)	23.00 (10.00, 54.00)
Age at HAE nC1-INH or AE-UNK diagnosis		
N	37	20
Mean (SD)	39.38 (17.84)	36.95 (15.49)
Median (range)	35.00 (12.00, 73.00)	32.50 (16.00, 80.00)
Time to diagnosis from onset of symptoms (in years)		
N	28	13
Mean (SD)	10.82 (13.38)	10.46 (16.81)
Median (range)	7.00 (0.00, 43.00)	2.00 (− 10.00, 50.00)
Ethnicity^a^		
Caucasian	35 (95%)	23 (100%)
Black	1 (3%)	0 (0%)
White South African	1 (3%)	0 (0%)
Unknown ethnicity	1 (3%)	0 (0%)
General population density		
Rural	12 (32%)	6 (26%)
Urban	25 (68%)	17 (74%)
Family history^a^		
Mother	16 (43%)	0 (0%)
Father	6 (16%)	0 (0%)
Sibling	19 (51%)	0 (0%)
Daughter	11 (30%)	0 (0%)
Other	11 (30%)	0 (0%)
No family history	0 (0%)	23 (100%)
Angioedema therapy prior to diagnosis^a^		
Antihistamines	22 (59%)	14 (61%)
Epinephrine	14 (38%)	8 (35%)
Corticosteroids	13 (35%)	9 (39%)
Omalizumab	1 (3%)	0 (0%)
Other therapies	8 (22%)	3 (13%)
Attack triggers^a^		
ACE inhibitors	2 (5%)	1 (4%)
Estrogens	13 (35%)	2 (9%)
Pregnancy	4 (11%)	0 (0%)
Stress	24 (65%)	6 (26%)
Infection	13 (35%)	4 (17%)
Trauma	11 (30%)	4 (17%)
Other trigger	14 (38%)	9 (39%)
Factor XII gene (1032C → A (Thr309Lys)) genetic testing results		
Negative	12 (32%)	1 (4%)
Not Tested	25 (68%)	22 (96%)
Factor XII gene (1032C → G (Thr309Arg)) genetic testing results		
Negative	13 (35%)	1 (4%)
Not tested	24 (65%)	22 (96%)
Angiopoetin -1 (ANGPT-1) genetic testing results		
Negative	8 (22%)	0 (0%)
Not tested	29 (78%)	23 (100%)
Plasminogen gene (PLG) genetic testing results		
Positive	2 (5%)	0 (0%)
Negative	9 (24%)	0 (0%)
Not tested	26 (70%)	23 (100%)

^a^Patients can have multiple values and may be included in more than one category, therefore, percentages may not add up to 100%. HAE nC1-INH, non-histaminergic hereditary angioedema with family history; AE-UNK, non-histaminergic hereditary angioedema without family history; SD, Standard deviation

Among patients with HAE nC1-INH, stress and estrogen therapy were the predominant triggers for angioedema attacks (65% and 35%, respectively; Table 1). It was also found that there were differences between patients with HAE nC1-INH and those with AE-UNK; with stress and estrogen therapy less likely to be a trigger for patients with AE-UNK (65% vs. 26%, p = 0.007, and 35% vs. 9%, p = 0.031 respectively, Table 2).

**Table 2:** Differences in attack characteristics and triggers between patients with HAE nC1-INH and AE-UNK before treatment

	HAE nC1-INH (N = 37)	AE-UNK (N = 23)	p-value
Average duration of attack in the 3 months before treatment (days)			0.124
N	24	9	
Mean (SD)	1.52 (1.61)	2.33 (1.68)	
Median (range)	1.00 (0.00, 7.00)	1.50 (0.50, 5.00)	
Number of attacks in the 3 months before treatment			0.190
N	26	10	
Mean (SD)	24.12 (28.89)	7.90 (10.25)	
Median (range)	10.50 (0.00, 90.00)	6.00 (1.00, 36.00)	
Site affected by attacks in the 3 months before treatment^a^			
Extremities	14 (38%)	3 (13%)	**0.045**
Face	10 (27%)	6 (26%)	1.000
GI system	21 (57%)	5 (22%)	**0.015**
Larynx	12 (32%)	5 (22%)	0.557
Lip	9 (24%)	4 (17%)	0.749
Tongue	7 (19%)	5 (22%)	1.000
Symptoms during attacks in the 3 months before treatment^a^			
Abdominal pain	18 (49%)	5 (22%)	0.056
Laryngeal edema	11 (30%)	5 (22%)	0.561
Skin swelling	16 (43%)	6 (26%)	0.271
Tongue swelling	9 (24%)	6 (26%)	1.000
Attack triggers^b^			
ACE inhibitors	2 (5%)	1 (4%)	1.000
Estrogens	13 (35%)	2 (9%)	**0.031**
Pregnancy	4 (11%)	0 (0%)	0.288
Stress	24 (65%)	6 (26%)	**0.007**
Infection	13 (35%)	4 (17%)	0.157
Trauma	11 (30%)	4 (17%)	0.366
Other trigger	14 (38%)	9 (39%)	1.000

Bolded values are statistically significant (p <0.05). ER, emergency room, GI, gastrointestinal; HAE nC1-INH, non-histaminergic hereditary angioedema with family history; AE-UNK, non-histaminergic hereditary angioedema without family history; SD, Standard deviation

^a^As every attack can affect more than one area, have more than one indication of severity, and be involved with or than one symptom, percentages may not add up to 100%

^b^Patients can have multiple attack triggers, and non-HAE related therapies and may be included in more than one category, therefore, percentages may not add up to 100%

### Treatment use

Among patients with HAE nC1-INH, 89% received on demand therapy at some point during the study period, versus 91% of patients with AE-UNK (Supplementary Table 1). The median (range) number of on demand treatments received by patients was 1 [1–3] for both patients with HAE nC1-INH and patients with AE-UNK (Supplementary Table 1). The most utilized on demand therapies for patients with HAE nC1-INH were intravenous pdC1-INH (59%) and icatibant (38%; Supplementary Table 1), and for patients with AE-UNK, they were also intravenous pdC1-INH (52%), and icatibant (48%; Supplementary Table 1).

**Table 3:** Attack characteristics in patients with HAE nC1-INH before and after HAE treatment initiation

	Before (N = 37)	After (N = 37)	p-value
Average duration of attack in the 3 months before/after treatment (days)			**0.001**
N	24	24	
Mean (SD)	1.52 (1.61)	0.86 (1.11)	
Median (Range)	1.00 (0.00, 7.00)	0.29 (0.02, 4.00)	
Number of attacks in the 3 months before/after treatment			**0.004**
N	26	28	
Mean (SD)	24.12 (28.89)	9.71 (12.49)	
Median (Range)	10.50 (0.00, 90.00)	6.00 (0.00, 60.00)	

Bolded values are statistically significant (p <0.05). SD, Standard deviation

**Fig. 1:**
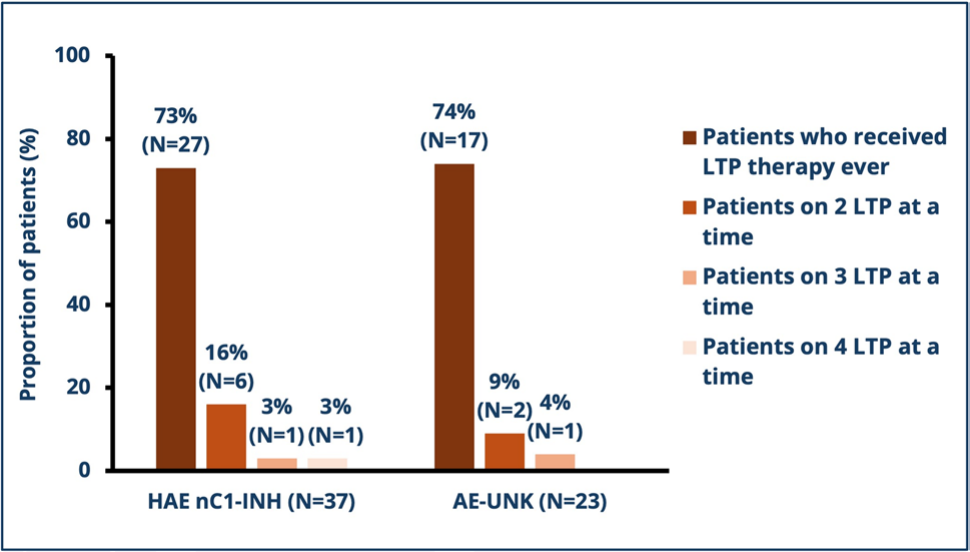
LTP treatment use. HAE nC1-INH, non-histaminergic hereditary angioedema with family history; AE-UNK, non-histaminergic hereditary angioedema without family history

In the context of LTP treatments, it was found that 22% of patients with HAE nC1-INH and 13% of patients with AE-UNK were taking 2 or more LTP treatment concurrently (Fig. 1). The LTPs most often prescribed for patients with HAE nC1-INH were subcutaneous pdC1-INH (43%) and tranexamic acid (41%). For patients with AE-UNK, the LTPs most often used were also subcutaneous pdC1-INH (39%) and tranexamic acid (35%; Supplementary Table 2).

### Clinical outcomes

Examination of clinical outcomes before and after treatment initiation demonstrated that for both patients with HAE nC1-INH and AE-UNK, following treatment initiation, duration and number of angioedema attacks decreased. For patients with HAE nC1-INH, it was found that the median (range) duration of attacks significantly decreased to 0.29 days (0.02–4.00) following treatment initiation, compared to 1.00 days (0.00–7.00) before treatment (p = 0.001, Table 3). There was also a significant decrease in the median (range) number of attacks in the three months after treatment initiation 6.00 (0.00–60.00) compared to the three months before treatment initiation 10.50 (0.00–90.00, p = 0.004, Table 3). Similarly, a decreasing trend in the duration and number of angioedema attacks before and after HAE treatment initiation was observed for patients with AE-UNK; however, these results were not significant.

Differences were observed between patients with HAE nC1-INH and AE-UNK in terms of the characteristics of angioedema attacks before and after treatment initiation. Comparing patients with HAE nC1-INH to patients with AE-UNK, in the three months before HAE treatment initiation, there were significantly fewer patients with AE-UNK experiencing angioedema attacks affecting their extremities (38% vs. 13%, p = 0.045) and GI system (57% vs. 22%, p = 0.015; Table 2). Similarly, in the three months after HAE treatment initiation, there were significantly fewer patients with AE-UNK experiencing angioedema attacks affecting their GI system (57% vs. 26%, p = 0.032, Table 4) compared to patients with HAE nC1-INH.

**Table 4:** Differences in attack characteristics between patients with HAE nC1-INH and AE-UNK after treatment initiation

	HAE nC1-INH (N = 37)	AE-UNK (N = 23)	p-value
Average duration of attack in the 3 months after treatment (days)			0.948
N	24	8	
Mean (SD)	0.86 (1.11)	1.28 (1.73)	
Median (range)	0.29 (0.02, 4.00)	0.58 (0.00, 4.00)	
Number of attacks in the 3 months after treatment			**0.044**
N	28	12	
Mean (SD)	9.71 (12.49)	6.08 (13.37)	
Median (range)	6.00 (0.00, 60.00)	2.00 (0.00, 48.00)	
Site affected by attacks in the 3 months after treatment^a^			
Extremities	9 (24%)	6 (26%)	1.000
Face	12 (32%)	7 (30%)	1.000
GI system	21 (57%)	6 (26%)	**0.032**
Larynx	9 (24%)	4 (17%)	0.749
Lip	12 (32%)	5 (22%)	0.557
Tongue	9 (24%)	5 (22%)	1.000
Symptoms during attacks in the 3 months after treatment^a^			
Abdominal pain	21 (57%)	6 (26%)	**0.032**
Laryngeal edema	7 (19%)	4 (17%)	1.000
Skin swelling	16 (43%)	7 (30%)	0.416
Tongue swelling	9 (24%)	6 (26%)	1.000

Bolded values are statistically significant (p <0.05). HAE nC1-INH, non-histaminergic hereditary angioedema with family history; AE-UNK, non-histaminergic hereditary angioedema without family history

^a^As every attack can affect more than one area, and be involved with or than one symptom, percentages may not add up to 100%

### Healthcare resource utilization

The median (range) number of primary care visits per year associated with HAE nC1-INH management in the first year following diagnosis was 3.0 (1.0–8.0, N = 9), and was 1.0 (1.0–1.0, N = 1) by year 7. Equally small patient numbers were observed for AE-UNK and results were similar (Supplementary Table 3).

## Discussion

This is the first study to evaluate the demographics, treatment response, and healthcare resource utilization among patients with HAE nC1-INH and AE-UNK, across Canada. The main findings of the study are (1) there is an average delay in diagnosis of more than 10 years for these conditions; (2) the frequency of angioedema attacks before starting LTP is high; and (3) despite a significant response to treatment, many patients continue to have frequent episodes.

The average age at diagnosis was 39.4 years for HAE nC1-INH and 37.0 years for AE-UNK, representing a > 10-year diagnostic delay from the first onset of symptoms. This delay underscores the current difficulties in making these diagnoses given the absence of confirmatory tests for most patients, rarity of the diseases, and requirement for highly specialized physician assessment. This could potentially be addressed by establishing consensus guidelines specifically for these conditions that describe diagnostic and therapeutic approaches based on the best available evidence and collective experience.

Prior to the initiation of HAE treatment, patients with both conditions were highly symptomatic. In the three months before treatment, the mean angioedema attack frequency was 24.1 and 7.9 for HAE nC1-INH and AE-UNK, respectively. The duration of attacks was 1.5 days for HAE nC1-INH and 2.3 days for AE-UNK. These data taken together with the diagnostic delay indicates a highly significant burden of disease in untreated patients.

Clinical outcomes after treatment initiation demonstrated that starting treatment decreased the duration and number of angioedema attacks, thus potentially supporting the use of preventative treatments such as pd-C1-INH and tranexamic acid for angioedema attacks in patients with HAE nC1-INH and AE-UNK. However, the mean number of attacks after treatment initiation was still high with an average of 9.7 attacks over three months, lasting an average of 0.86 days, in HAE nC1-INH. Additionally, 22% of patients with HAE nC1-INH and 13% of patients with AE-UNK were receiving more than one LTP treatment concurrently, suggesting poor efficacy of monotherapy in many patients, with clinicians combining LTP in an effort to improve control. This suggests a significant unmet need for effective preventative treatments for these patients.

Baseline demographics and clinical characteristics were in keeping with previously published studies investigating patients with HAE nC1-INH [1, 5, 13, 14]. For both HAE nC1-INH and AE-UNK, patients were predominantly women and the age at first symptom/attack was 21.5 (5.0–57.0) and 23.0 (10.0–50.0) years, respectively. AE-UNK is generally thought to present similarly to HAE nC1-INH [5], and this may be because they represent the same disease in certain patients. At least some cases of AE-UNK are likely the result of de novo mutations, which is seen in ~ 25% of patients with HAE C1-INH, and thus these patients would be reclassified as HAE nC1-INH when the next generation is found to be symptomatic. We thus investigated the differences between patients with HAE nC1-INH and AE-UNK. Anacdotally, it is thought that patients with HAE nC1-INH and AE-UNK both equally experience stress as the predominant attack trigger. However, interestingly, in this study it was found that among patients with HAE nC1-INH, 65% experienced stress as the predominant trigger for angioedema—similar to a recent study of 295 patients with HAE nC1-INH in which stress was identified as the second most common trigger for angioedema attacks, affecting 59.6% of patients [14]—however this was significantly different (p = 0.007) to patients with AE-UNK, for whom 26% experienced stress as the attack trigger.

Furthermore, in the three months before HAE treatment initiation, there were significantly fewer patients with AE-UNK experiencing angioedema attacks affecting their extremities and GI system compared to patients with HAE nC1-INH. Following treatment initiation, significantly fewer patients with AE-UNK experienced angioedema attacks affecting their GI system and fewer patients experienced abdominal pain. Interestingly, this is the first time that significant differences for the symptomatic presentation of angioedema attacks between patients with HAE nC1-INH and AE-UNK have been observed. Caution is needed, however, as a trial of high-dose antihistamine therapy is generally recommended prior to diagnosing these conditions, particularly AE-UNK in which genetic testing and family history is negative, but a substantial minority of patients did not receive antihistamines, so these differences could be accounted for by inclusion of patients with mast cell mediated angioedema in the AE-UNK group [15]. Further studies using rigorously defined patient populations, and appropriate genetic testing are needed to confirm these observations.

The study has important limitations—the diagnosis of HAE nC1-INH or AE-UNK was established by individual clinicians, not using a specific set of standardized inclusion criteria. In particular, some were not treated with antihistamines and most were not treated with omalizumab prior to establishing the diagnosis, so patients with mast cell mediated angioedema may have been included. However, the presented data reflect current clinical practice in Canada. Data were collected from specialized centers, which may have introduced bias by including more severely affected patients. Given the retrospective design using data extracted there are inherent limitations due to the availability and accuracy of data. Certain variables are not consistently documented in medical notes, such as the details of each angioedema attack and dates of each healthcare resource utilization. As many angioedema attacks and visits with external healthcare providers related to HAE occur outside of the HAE specialist’s clinic settings, these data are only collected if a patient reports them to their treating HAE specialist. The number of included patients was small, consistent with the rarity of the conditions. Despite these limitations, this is the only real-world study examining outcomes in HAE nC1-INH and AE-UNK, across Canada.

## Conclusions

There is an average diagnostic delay of more than a decade in patients with HAE nC1-INH and AE-UNK, and these patients often have frequent angioedema episodes without treatment. Presently available treatment options in Canada for these patients appear to improve angioedema frequency and duration, but patients remain significantly affected. Future prospective studies would be helpful to better understand the clinical manifestations, disease burden, and treatment responses in these diseases.

## Supplementary Information

Additional file 1.

## Data Availability

The data underlying this article cannot be shared publicly due to the privacy of individuals. The data presented in this study may be available on reasonable request from the corresponding author.

## Abbreviations

AB: Alberta
ACE: Angiotensin-converting-enzyme
AE-UNK: Idiopathic angioedema of unknown etiology
ANGPT-1: Angiopoetin-1
BC: British Columbia
cERF: Electronic case report form
EHR: Electronic health records
ER: Emergency room
GI: Gastrointestinal
GPP: Guidelines for good pharmacoepidemiology
HAE: Hereditary angioedema
HAE nC1-INH: Hereditary angioedema with normal C1-inhibitor function
LTP: Long-term prophylaxis
MB: Manitoba
NA: Not applicable
ON: Ontario
pdC1-INH: Plasma-derived C1 inhibitor
PLG: Plasminogen gene
QC: Québec
RWE: Real-world evidence
SD: Standard deviation

## References

[1] Jones DH, Bansal P, Bernstein JA, Fatteh S, Harper J, Hsu FI, et al. Clinical profile and treatment outcomes in patients with hereditary angioedema with normal C1 esterase inhibitor. World Allergy Organ J. 2022;15(1): 100621. 35145604 10.1016/j.waojou.2021.100621 PMC8804245

[2] Busse PJ, Christiansen SC. Hereditary angioedema. NEJM. 2020;382(12):1136–48. 32187470 10.1056/NEJMra1808012

[3] Bouillet L, Boccon-Gibod I, Launay D, Gompel A, Kanny G, Fabien V, et al. Hereditary angioedema with normal C1 inhibitor in a French cohort: clinical characteristics and response to treatment with icatibant. Immun Inflamm Dis. 2017;5(1):29–36. 28250922 10.1002/iid3.137 PMC5322159

[4] Veronez CL, Csuka D, Sheikh FR, Zuraw BL, Farkas H, Bork K. The expanding spectrum of mutations in hereditary angioedema. J Allergy Clin Immunol Pract. 2021;9(6):2229–34. 33746090 10.1016/j.jaip.2021.03.008

[5] Hinther K, Kalincinsky C. Review of type III non-hereditary angioedema patients in Manitoba. Res Sq. 2020. 10.21203/rs.3.rs-76142/v1.

[6] Reshef A, Buttgereit T, Betschel SD, et al. Definition, acronyms, nomenclature, and classification of angioedema (DANCE): AAAAI, ACAAI, ACARE, and APAAACI DANCE consensus. J Allergy Clin Immunol. 2024. 10.1016/j.jaci.2024.03.024. 38670233 10.1016/j.jaci.2024.03.024

[7] Sinnathamby ES, Issa PP, Roberts L, Norwood H, Malone K, Vemulapalli H, et al. Hereditary angioedema: diagnosis, clinical implications, and pathophysiology. Adv Ther. 2023;40(3):814–27. 36609679 10.1007/s12325-022-02401-0 PMC9988798

[8] Betschel S, Badiou J, Binkley K, Borici-Mazi R, Hébert J, Kanani A, et al. The International/Canadian hereditary angioedema guideline. Allergy Asthma Clin Immunol. 2019;15(1):72. 31788005 10.1186/s13223-019-0376-8 PMC6878678

[9] Bork K, Wulff K, Witzke G, Hardt J. Hereditary angioedema with normal C1-INH with versus without specific F12 gene mutations. Allergy. 2015;70(8):1004–12. 25952149 10.1111/all.12648

[10] McKibbin L, Barber C, Kalicinsky C, Warrington R. Review of the Manitoba cohort of patients with hereditary angioedema with normal C1 inhibitor. Allergy Asthma Clin Immunol. 2019;15(1):66. 31749860 10.1186/s13223-019-0381-y PMC6852747

[11] Buttgereit T, Nicola S, Vera C, Brussino L, Maurer M, Magerl M. Significant response to berotralstat in 3 patients with hereditary angioedema of unknown origin. J Allergy Clin Immunol Pract. 2023;11(12):3804-3807.e2. 37598729 10.1016/j.jaip.2023.08.018

[12] Adatia A, Ritchie B. Successful use of lanadelumab in a patient with hereditary angioedema with normal C1 inhibitor and negative genetic testing. J Allergy Clin Immunol Glob. 2023;2(2): 100087. 37780787 10.1016/j.jacig.2023.100087 PMC10509950

[13] Jones D, Zafra H, Anderson J. Managing diagnosis, treatment, and burden of disease in hereditary angioedema patients with normal C1-esterase inhibitor. J Asthma Allergy. 2023;16:447–60. 37124440 10.2147/JAA.S398333 PMC10132308

[14] Fragnan NT, Veronez C, Moreno A, Arruda LKP, Goncalves RF, Valle S, et al. Treatment of patients with hereditary angioedema with normal C1 inhibitor: evaluation of 295 patients. J Allergy Clin Immunol. 2019;143(2):AB40.

[15] Buttgereit T, Fijen LM, Vera C, Bergmann KC, Maurer M, Magerl M. Case report: recurrent angioedema: diagnosing the rare and the frequent. Front Med (Lausanne). 2022;9:1048480. 36530887 10.3389/fmed.2022.1048480 PMC9756157

