# Supplementary material for: Real-world outcomes of patients with hereditary angioedema with normal C1-inhibitor function and patients with idiopathic angioedema of unknown etiology in Canada
Source: Allergy Asthma Clin Immunol. 2024 Sep 27;20:50. doi: 10.1186/s13223-024-00910-x (PMC11438182; doi:10.1186/s13223-024-00910-x)
Supplement: Supplementary file 1 — Additional file 1. [file 13223_2024_910_MOESM1_ESM.docx]

**Supplementary Material**

**Supplementary Table 1. On demand therapy use among patients with HAE nC1-1NH and AE-UNK**

|  | **HAE nC1-INH (N=37)** | **AE-UNK (N=23)** |
| --- | --- | --- |
| **Number of patients who received on demand therapy ever** | 33 (89%) | 21 (91%) |
| **Number of on demand treatments received by patients** |  |  |
| N | 33 | 21 |
| Mean (SD) | 1.36 (0.60) | 1.29 (0.56) |
| Median (Range) | 1.00 (1.00, 3.00) | 1.00 (1.00, 3.00) |
| **Type of on demand therapy received by patients^a^** |  |  |
| pdC1-INH (intravenous) | 22 (59%) | 12 (52%) |
| pdC1-INH (subcutaneous) | 0 (0%) | 1 (4%) |
| Icatibant | 14 (38%) | 11 (48%) |
| Tranexamic acid | 4 (11%) | 1 (4%) |

^a^Patients may receive more than one type of on-demand therapy and may be included in more than one category, therefore, percentages may not add up to 100%. HAE nC1-INH, non-histaminergic hereditary angioedema with family history; AE-UNK, non-histaminergic hereditary angioedema without family history; pdC1-INH, plasma derived C1-inhibitor; SD, Standard deviation.

**Supplementary Table 2. LTP treatment use among patients with HAE nC1-1NH and AE-UNK**

|  | **HAE nC1-INH (N=37)** | **AE-UNK (N=23)** |
| --- | --- | --- |
| **Number of patients who received LTP therapy ever** | 27 (73%) | 17 (74%) |
| **Type of LTP received by patients^a^** |  |  |
| Lanadelumab | 1 (3%) | 0 (0%) |
| pdC1-INH (intravenous) | 7 (19%) | 5 (22%) |
| pdC1-INH (subcutaneous) | 16 (43%) | 9 (39%) |
| pdC1-INH (unidentified) | 0 (0%) | 1 (4%) |
| androgenic steroids | 3 (8%) | 1 (4%) |
| tranexamic acid | 15 (41%) | 8 (35%) |
| progestin | 1 (3%) | 2 (9%) |
| Cetirizine | 1 (3%) | 0 (0%) |
| Singulair | 1 (3%) | 0 (0%) |
| Sulfasalazine | 0 (0%) | 1 (4%) |
| **Number of patients on 2 LTP treatments at a time** | 6 (16%) | 2 (9%) |
| **Number of patients on 3 LTP treatments at a time** | 1 (3%) | 1 (4%) |
| **Number of patients on 4 LTP treatments at a time** | 1 (3%) | 0 (0%) |
| **2 LTP treatment combinations** |  |  |
| pdC1-INH (intravenous), androgenic steroids | 1 | 0 |
| pdC1-INH (subcutaneous), tranexamic acid | 2 | 0 |
| pdC1-INH (intravenous), tranexamic acid | 3 | 0 |
| pdC1-INH (subcutaneous), sulfasalazine | 0 | 1 |
| Progestin, tranexamic acid | 0 | 1 |
| **3 LTP treatment combinations** |  |  |
| pdC1-INH (intravenous), androgenic steroids, tranexamic acid | 1 | 1 |
| **4 LTP treatment combinations** |  |  |
| Cetirizine, pdC1-INH (subcutaneous), singulair, tranexamic acid | 1 | 0 |

^a^Patients may receive more than one type of LTP treatment and may be included in more than one category, therefore, percentages may not add up to 100%. HAE nC1-INH, non-histaminergic hereditary angioedema with family history; AE-UNK, non-histaminergic hereditary angioedema without family history; pdC1-INH, plasma derived C1-inhibitor; SD, Standard deviation

**Supplementary Table 3. Healthcare utilization among patients with HAE nC1-INH and AE-UNK following diagnosis**

|  | **Year 1** | **Year 2** | **Year 3** | **Year 4** | **Year 5** | **Year 6** | **Year 7** | **Year 8** | **Year 9** | **Year 10** |
| --- | --- | --- | --- | --- | --- | --- | --- | --- | --- | --- |
| **Number of primary care visits per year associated with HAE nC1-INH management** |  |  |  |  |  |  |  |  |  |  |
| N^a^ | 9 | 8 | 7 | 6 | 6 | 2 | 1 | 2 | 0 | 0 |
| Mean (SD) | 3.78 (2.22) | 2.88 (1.89) | 2.14 (1.68) | 2.50 (1.87) | 1.17 (0.75) | 1.50 (0.71) | 1.00 (NA) | 1.00 (0.00) | NA | NA |
| Median (Range) | 3.00 (1.00, 8.00) | 2.00 (1.00, 6.00) | 1.00 (1.00, 5.00) | 2.00 (1.00, 6.00) | 1.00 (0.00, 2.00) | 1.50 (1.00, 2.00) | 1.00 (1.00, 1.00) | 1.00 (1.00, 1.00) | NA | NA |
| **Number of primary care visits per year associated with AE-UNK management** |  |  |  |  |  |  |  |  |  |  |
| N^b^ | 2 | 2 | 2 | 2 | 2 | 2 | 2 | 2 | 2 | 2 |
| Mean (SD) | 2.00 (0.00) | 1.50 (0.71) | 0.50 (0.71) | 1.00 (0.00) | 1.50 (2.12) | 0.50 (0.71) | 1.50 (0.71) | 0.50 (0.71) | 0.50 (0.71) | 0.50 (0.71) |
| Median (Range) | 2.00 (2.00, 2.00) | 1.50 (1.00, 2.00) | 0.50 (0.00, 1.00) | 1.00 (1.00, 1.00) | 1.50 (0.00, 3.00) | 0.50 (0.00, 1.00) | 1.50 (1.00, 2.00) | 0.50 (0.00, 1.00) | 0.50 (0.00, 1.00) | 0.50 (0.00, 1.00) |

^a^N is the number of times the clinician recorded the number of healthcare visit (including records of 0 visits).

HAE nC1-INH, non-histaminergic hereditary angioedema with family history; ; NHAE nC1-INH, non-histaminergic hereditary angioedema without family history, SD, Standard deviation
